# The Endothelial Glycocalyx and Neonatal Sepsis

**DOI:** 10.3390/ijms24010364

**Published:** 2022-12-26

**Authors:** Ahlam Fatmi, Wiam Saadi, Jesús Beltrán-García, José Luis García-Giménez, Federico V. Pallardó

**Affiliations:** 1 INCLIVA Health Research Institute, Mixed Unit for Rare Diseases INCLIVA-CIPF, 46010 Valencia, Spain; 2 Department of Biology, Faculty of Nature, Life and Earth Sciences, University of Djillali Bounaama, Khemis Miliana 44225, Algeria; 3 Center for Biomedical Network Research on Rare Diseases (CIBERER), Institute of Health Carlos III, 46010 Valencia, Spain; 4 Department of Physiology, Faculty of Medicine and Dentistry, University of Valencia, 46010 Valencia, Spain; 5 Department of Medicine, Division of Regenerative Medicine, University of California, San Diego, CA 92093, USA

**Keywords:** biomarkers, endothelium, endothelial glycocalyx, neonatal sepsis, sepsis

## Abstract

Sepsis carries a substantial risk of morbidity and mortality in newborns, especially preterm-born neonates. Endothelial glycocalyx (eGC) is a carbohydrate-rich layer lining the vascular endothelium, with important vascular barrier function and cell adhesion properties, serving also as a mechano-sensor for blood flow. eGC shedding is recognized as a fundamental pathophysiological process generating microvascular dysfunction, which in turn contributes to multiple organ failure and death in sepsis. Although the disruption of eGC and its consequences have been investigated intensively in the adult population, its composition, development, and potential mechanisms of action are still poorly studied during the neonatal period, and more specifically, in neonatal sepsis. Further knowledge on this topic may provide a better understanding of the molecular mechanisms that guide the sepsis pathology during the neonatal period, and would increase the usefulness of endothelial glycocalyx dysfunction as a diagnostic and prognostic biomarker. We reviewed several components of the eGC that help to deeply understand the mechanisms involved in the eGC disruption during the neonatal period. In addition, we evaluated the potential of eGC components as biomarkers and future targets to develop therapeutic strategies for neonatal sepsis.

## 1. Introduction

Neonatal sepsis is a life-threatening condition in newborns. It is associated with a high risk of mortality, serious disability in survivors, and psychological problems, and thus responsible for a severe burden on healthcare services [1,2,3]. Importantly, neonatal sepsis in premature newborns is considered a rare disease (ORPHA:90051). Among the different research areas explored in sepsis, vascular endothelium is considered one of the most important organs affected during the initial onset of sepsis, particularly due to its systemic involvement, and its role in inflammation and in the coagulation process. Endothelial injury increases blood vessel permeability, vessel diameter, and fluid leakage from blood vessels into the interstitial space. Induced hypovolemia affecting tissue perfusion pressure, decrease in oxygen delivery, and hypotension development within a few hours, lead neonates to septic shock [4,5].

Danielli (1940) was the first who described this protein layer on the endothelium [6]. Glycocalyx, from the Greek ‘sugar coat’ (from glykys, sweet and kalyx husk) [7], is a carbohydrate-rich layer (mainly composed of proteoglycans and glycosaminoglycans), which is found surrounding the membrane of many cell types, covering the luminal surface of vascular endothelial cells (EC) [8,9] and comprises membrane-attached proteoglycans, glycosaminoglycan chains, glycoproteins, and adherent plasma proteins, of which syndecans and glypicans are the most prevalent [10]. Endothelial glycocalyx (eGC) thickness differs with location and vascular bed, and can range from 0.1 to 4.5 µm [11,12]. According to a study by Xia et al. using confocal microscopy, the culture of human cerebral microvascular endothelial cells was determined: the thickness of heparan sulfate elements was 1.53 μm and hyaluronic elements was 2.23 μm, comparable to those observed at the microvessels [13]. In rat myocardial capillaries the thickness of eGC ranged from 0.2 to 0.5 µm and its degradation induced myocardial tissue edema [14]. These measures can differ in rat mesenteric capillaries and post-capillary venules (0.9 ± 0.1 and 1.2 ± 0.3 μm, respectively) [15]. In different models, it was found that under inflammatory conditions, the eGC is heavily damaged and takes about 5–7 days to initiate the recovery, after which endothelial and microcirculatory functions can be restored [16]. However, the visualization of the eGC to determine its composition and thickness has proven difficult due to its extremely delicate and rapidly disrupted structure [17].

Glycocalyx in newborns takes special relevance because it is one of the dominant components of the mucosal immune system [18], given that it is one of the earliest sites of injury during inflammation [19]. eGC assessment in pediatric clinical studies is based mainly on two different approaches which provide only indirect information about the eGC. The first approach measures the levels of eGC-detached components in plasma/serum and urine, such as syndecan-1 (SDC-1), hyaluronan (HA), heparan sulphate (HS), and heparan sulphate chondroitin (CS). The second approach uses the video microscopic assessment of the eGC in the microcirculation vessels [20].

Currently, there are scarce studies on the eGC in neonatal sepsis. In the present review, our aim is to highlight the most important contributions to provide a state-of the-art glycocalyx research in neonatal sepsis. Accordingly, we reviewed literature related with eGC and sepsis from 1999 to 2022, with special emphasis on those papers published in the last five years. From these published papers we have included the largest number of parameters that help, not only to deeply understand the mechanisms of eGC disruption in the neonatal period, but also to evaluate the potential of eGC components as biomarkers and identify future targets to develop potential therapeutic strategies for neonatal sepsis.

## 2. Structure of Endothelial Glycocalyx

**Figure 1:**
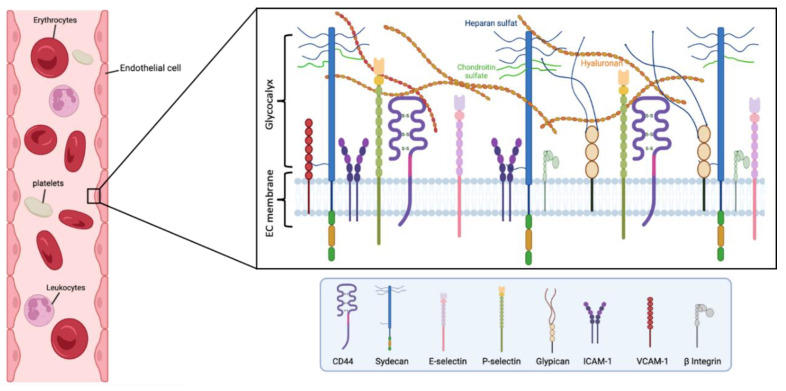
Main components of the human endothelial glycocalyx. The eGC consists of proteoglycans, glycoproteins, and glycosaminoglycans, associated with plasma proteins. Among others, CD44 is a cell-surface receptor for HA, it plays an important role in cell proliferation, and migration, and participates in vascular barrier integrity via the regulation of CD31 expression. β Integrin participates in leukocyte homing and subsequent diapedesis, and platelet interactions via a link to collagen, fibronectin, and laminin in the subendothelial matrix. P-selectin and E-selectin are implicated in the ‘tethering’ and rolling’ of leukocytes in stimulated‘ endothelial cells. Particularly, E-selectin needs direct stimulation by cytokines such as IL-1, TNF-α, or LPS for production and surface expressio. Syndecans and glypicans are proteoglycans that contain GAG chains such as HS and CS. Moreover, syndecans represent the principal effector in cell adhesion or shape changes by their interaction with the cytoskeleton, while the glypicans have a role in flow-induced endothelial NO synthase activation. The intercellular adhesion glycoprotein (ICAM-1) is expressed in the endothelial cell surface participating in cell-to-cell interactions and facilitates leukocyte endothelial transmigration. VCAM-1 is a glycoprotein which contributes to the cell adhesion of molecules to the endothelium. Figure created with BioRender.com.

The thickness of the eGC is an essential parameter in measuring the functional performance of this layer in the blood vessels, where this thickness changes according to the organ in which the endothelium is present. eGC is found in continuous and fenestrated capillaries, in which it is thicker than sinusoid capillaries (Figure 1) [21]. eGC appears in continuous-type heart capillaries in moss- or broccoli-like structures covering the entire luminal endothelial cell surface. In the surface of renal podocytes, eCG is located near to occlude the endothelial pores of the fenestrated capillaries. In the sinusoid capillaries, it is in the liver, and in hematopoietic organs, covering both the luminal and the opposite side. Particularly, in the liver, the eGC is located between a hepatocyte and a sinusoid, known as the space of Disse [22].

Generally, eGC appears as a gel-like layer of glycoproteins covering the luminal surface of the capillary endothelium. eGC has an intricate architecture of different components incorporating either plasma- or endothelium-derived soluble molecules. eGC is formed by soluble plasma components linked together either directly or via proteoglycans and/or glycosaminoglycans (GAG) [23]. The components of proteoglycans entail a core protein attached to GAG chains [21], where HS comprises 50–90% of proteoglycans. Small proteogylcans such as syndecans and glypicans bind to the endothelial membrane, and other proteoglycans such as perlecan, versican, decorin, biglycan, and mimecan can be secreted as soluble proteoglycans, which can also bind to multiple GAG side chains (e.g., HS and CS, which bind to syndecans, glypicans, and perlecan; CS and dermatan sulfate, which bind to versican, decorin, and biglycan; and mimecan, which binds to keratan sulfate). These components are responsible for a charge-negative mesh through capillaries, facilitating a frictionless blood flow [24]. Importantly, the parameters of rheology in vessels continuously affect the thickness of the glycocalyx by affecting the composition and structure, through enzymatic or shear-induced shedding processes. Notably, the dynamic balance between biosynthesis and shedding makes it quite complicated to describe properly the geometrical disposition and distribution of the eGC [23]. 

Several components of the glycocalyx, including syndecans, HS, and HA are altered in cases of ischemia, hypoxia, sepsis, atherosclerosis, renal disease, diabetes, and several viral infections [25,26]. This alteration induces deleterious effects on the eGC and therefore in the endothelium, leading to dysfunction of microcirculatory with subsequent organ ischemia, and finally consequent organ damage.

## 3. Physiological Function of the Endothelial Glycocalyx

Under physiological conditions, there is a dynamic balance between the biosynthesis of new GAGs and the shear-dependent removal of different components of the eGC. This gives the eGC high structural stability, working as a vasoprotective nanobarrier against vascular leakage and adhesion, and avoiding vessel inflammation [8,27]. Importantly, the eGC can respond to environmental changes by adapting its nanomechanical properties [28]. It is known that the alterations in the hydrostatic pressure, the flow rate, and the influences of the gradient concentration in blood vessels, play an important role in the permeability properties of the eGC [7]. Although the eGC has a mince layer, it has a prominent enzyme regulatory system, which can participate in modulating the expression of functional mediators, that ultimately are involved in the blood vessel barrier integrity (i.e., albumin, antithrombin, HS, and antioxidants) [29]. That means eGC is a physical transducer, which can mediate shear-dependent endothelial responses and act as a selective plasma-filtering system of different macromolecules. In addition, eGC conserves binding sites for endothelial growth factors, fibroblast growth factor, lipoprotein lipase, superoxide dismutase, and antithrombin III [19], which in turn contributes to hydrolyzing triglycerides, balancing oxidative stress, and regulating oxidative stress, respectively. Finally, the eGC also regulates the leukocyte-endothelial adhesion process [5,30], a crucial event in immune responses, and especially important during sepsis [31].

## 4. Endothelial Glycocalyx in Newborns

Few studies have explored the eGC in neonates or during the prenatal period, mainly due to the difficulties of acquiring samples in newborns. Despite this, we already know that the eGC plays an important role in the formation and maturation of the vascular system [20]. According to Ank et al., levels of placental proteoglycans such as glypican-1, glypican-3, and syndecan-1 do not differ in the umbilical cord blood from different gestational age groups [32]. 

In the fetal period, SDC-1 (an eGC breakdown marker), HS, and HA concentrations were increased in the serum of patients with HELLP (hemolysis, elevated liver enzymes, and low platelets) syndrome compared to normal pregnancy at similar gestational stages [33]. However, the concentrations of these molecules in the serum of pregnant women after 20 weeks were not correlated with the later development of gestational diabetes mellitus [34]. In another study performed by Kornacki et al., blood samples from three groups of pregnant women (first group in the third trimester with early-onset preeclampsia (*n* = 20), second group with late-onset preeclampsia (*n* = 20), and third group of normal pregnancy (*n* = 20)). Authors demonstrated a higher degree of glycocalyx degradation in early-onset preeclampsia patients than in those with the late-onset condition. These differences were associated with both HA and SDC-1 concentrations. In particular, HA levels were found to increase, and SDC-1 levels were decreased in preeclampsia patients compared to the control group [35]. Interestingly, it was found differences in flucosylated glycan levels between early and late onset fetal growth restriction (FGR). The increase of flucosylated glycan was associated with a severe pathological state, villous maturation disorders, and hypoxia adaptation, via changes in the cell proliferation cycle and the induction of angiogenesis [36]. 

In another study, glucidic sequences were characterized using lectin-binding experiments which evaluated the distribution and modulation of eGC on the surface of diverse blood vessels in adult and newborn pigs. These studies showed that the modulation of the EC surface sugar residues and von Willebrand factor (vWF) expression levels differ between newborn and mature pig ECs [37].

In 2015, Nussbaum et al. described the presence of the local loss of the eGC associated with microvascular perfusion in newborns after surgery with cardiopulmonary bypass [38]. In cardiac surgery patients, even baseline plasma concentrations of glycocalyx degradation products varied greatly [39]. Increased SDC-1 levels were found in patients who had undergone cardiothoracic surgery. The authors previously showed that plasma SDC-1 baseline concentrations in the neonate trial did not differ between the control (*n* = 20) and methylprednisolone (MP) group (*n* = 20). At later time points, SDC-1 significantly increased in the MP group at 30 min after cardiopulmonary bypass and continued rising up to 6 h after surgery. SDC-1 levels in the placebo-controlled continued increasing almost four-fold from the baseline concentration 6 h after surgery. Consequently, in neonates having undergone cardiothoracic surgery, eGC shedding can be reduced by the administration of a hefty dose of the corticosteroid hormone methylprednisolone [40], probably via the reduction of the systemic inflammatory response after cardiothoracic surgery.

Most microvascular abnormalities can cause alterations in the glycocalyx, which are sometimes reflected by increased red blood cell (RBC) penetration [41]. The perfused boundary region (PBR) is the permeable part of the eGC that allows the flow of erythrocytes to move nearer the endothelial surface, which increases the PBR, indicating a decrease in glycocalyx thickness [42]. Using Sidestream Dark Field (SDF) imaging for PBR measurement in the cutaneous microcirculation, Puchwein-Schwepcke et al. showed that the eGC in preterm (*n* = 39) and term (*n* = 85) neonates depends on the gestational age at birth. Intriguingly, the eGC dimension was shown to correlate inversely with gestational age. The thickest eGC was seen in the most immature neonates (reflected by low PBR values) [43]. In healthy mature newborns, PBR at 3 days of age was 2.14 ± 0.25 μm [43], while in adult human PBRs sublingual blood vessels were grouped into diverse diameters (5–9 μm, 10–19 μm, and 20–25 μm) [44].

In a prospective cohort study performed in 27-term infants and children (aged 5 days to 57 months old) following cardiopulmonary bypass surgery, a correlation between plasma concentration of HS and metabolic acidosis was found. In addition, the cleavage of HS was correlated with renal dysfunction, capillary leak, and global markers of cardiovascular dysfunction [45]. 

All these data point out the important role of eGC in neonatal development and demonstrate how eGC disturbances can produce pathological clinical phenotypes in neonates. 

In this regard, since sepsis is one of the most life-threatening conditions in newborns, considering the relevant role of the eGC in the formation and maturation of the vascular system in neonates, it seems especially important to elucidate the molecular mechanisms that are leading to the damage and shedding of the eGC during neonatal sepsis, potentially producing long-term vascular comorbidities in newborns.

## 5. Endothelial Glycocalyx and Sepsis

Before focusing on the role of the glycocalyx in sepsis, it is worth pointing out that glycocalyx is not only related to sepsis, but also has an important role in other diseases due to its role in the vascular circulation [46]. The pathological process associated with sepsis involves an abnormal immune response, with deviance in pro- and anti-inflammatory cytokine production, resulting potentially in significant multiorgan damage and dysfunction [47,48]. In the inflammatory process, the proinflammatory cytokines such as tumor necrosis factor (TNF)-α and IL-1α insulat eGC, leading to an increased leukocyte adhesion [27]. Moreover, losing the eGC contributes to edema formation [49]. In this regard, in vitro exposition of eGC to endotoxin, TNFα, Angiopoietin-2, or thrombin (all molecules released during sepsis) induced eGC damage [24]. Similarly, an experimental endotoxemia model using LPS showed the disruption of the eGC. In fact, cells appeared to be peeling off and clumping, which was associated with increased serum concentrations of SDC-1 in the first 24 h [50]. Enzymes such as metalloproteinases, heparanase, and hyaluronidase also participate in glycocalyx degradation. Besides pro-inflammatory and anti-inflammatory hyperactivated responses, sepsis is also characterized by a catabolic state of proteins, lipids, and carbohydrate consumption. In this regard, the endothelial dysfunction induced by LPS is associated with an increase in glycan production and fatty acid metabolism, accompanied by eGC loss, which induces an increment of 60% in the endothelial permeability. Moreover, data obtained from human sepsis studies suggest an increase in glycan synthesis, which seems to be one of the most affected metabolic pathways, especially in non-survivors [51]. Reactive oxygen species (ROS) and proinflammatory cytokines are implicated in the activation of sheddase enzymes that cleave extracellular portions of transmembrane proteins, which in turn contribute to the release of soluble ectodomains in transmembrane proteins from the cell surface. This degradation of transmembrane proteins causes an increased vascular permeability, dysregulation of vascular homeostasis, and microvascular thrombosis, which finally increases the leukocyte adhesion. 

An important clinical finding is a correlation between organ dysfunction, severity, and mortality in sepsis with circulatory levels of eGC components [49,52]. The increase in the concentration of GAGs proteins in the plasma of sepsis patients was notable, mainly in those patients who finally die. Moreover, the septic patients who died also showed the highest concentration of GAGs as well as the elevation of SDC-1 levels, which correlated with the SOFA score [53]. Several studies reported the ability of matrix metalloproteinases (MMP) to cleave syndecans, an essential component of proteoglycans. For example, MMP-9, which commonly causes SDC-1 degradation, or MMP-2, that induces the cleavage of SDC-4 [54]. It seems that the action of MMPs is facilitated by the loss of HS. However, neither exogenous HS nor heparin showed any effect on shedding, suggesting that HS must be bound to the core protein to control the sheddase of syndecans, or mediate the synthesis of core proteins [55].

In addition, the cleavage of eGC can be induced by hyaluronidase, which produces the conversion of high molecular hyaluronic acid into low molecular weight hyaluronic acid fragments [56], a process which stimulates ROS production during phagocytosis [57]. Furthermore, low molecular hyaluronic acid fragments produce the expression of vascular cell-adhesion molecule -1 (VCAM-1) and intercellular-adhesion molecule -1 (ICAM-1), resulting in increased macrophage activity, as well as inflammation and endothelial cell damage [57]. These processes may be modulated by the activation of extracellular heparanases by proinflammatory cytokines [54]. Particularly in humans, the endo-β-glucuronidase Heparanase-1 (Hpa-1) is decreased during sepsis and has been proposed as a biomarker to predict the risk of sepsis in adults [58]. Hpa-1 can cleave HS side chains from their proteoglycans, inducing the liberation of circulating HS-fragments, thus contributing to inflammation [59].

A disintegrin and metalloproteinases (ADAMs) are proteins that target both inflammatory and adhesion molecules. In this regard, the inducible form of ADAM17, which cleaves SDC-4, targets cell receptors for IL-6, TNF, and cell adhesion molecules (i.e., ICAM-1, VCAM-1, and L-selectin) expressed by leukocytes and endothelial cells [51]. The protease ADAM13 cleaves the vWF and contributes to microvascular thrombosis, microvascular ischemia, and organ failure. Interestingly, it has been observed a correlation between ADAM13 levels and sepsis severity and outcomes in pediatric patients. [60].

Moreover, when the expression of endogenous antagonist angiopoietin-2 (Ang-2) is increased, it displaces ang-1 from the endothelium-stabilizing receptor Tie2 in sepsis, inducing heightened heparanase expression, thus stimulating pathogenic sheddase of the endothelial glycocalyx [30,61]. In addition, Tie2 activation seems to accelerate eGC recovery, so it can set the bases of a new therapeutic strategy against sepsis [61]. Mechanistically, Tie2 activation can suppress the specific effect of enzyme heparanase and avoid the breakdown of HS [61,62,63]. Furthermore, in vitro experiments have demonstrated that after the addition of MCTR1 (maresin [macrophage mediator in resolving inflammation] conjugates in tissue regeneration 1) to the culture medium of EC challenged with LPS for 6 h, MCTR1 can inhibit HS degradation via the downregulation of heparanase protein expression [64]. Notably, the expression of adhesion molecules in neonatal EC is lower. Moreover, it has been demonstrated that neonatal EC has a low ability to detoxify ROS compared to the adult EC [65]. In addition, after LPS challenge of HUVECS from preterm neonates, altered levels of inducible E-selectin, ICAM-1, and VCAM-1 were found [66], pointing out the importance of eGC maintaining vascular homeostasis. 

## 6. Biomarkers of the Endothelial Glycocalyx in Sepsis

The study of biomarkers in sepsis may be focused to generate new tools for early detection, stratification, and prognosis of poor outcomes. The heterogeneous and complex features of sepsis is contributing to different research initiatives to provide new perspectives in biomedical research. Several clinical signs such as uncontrolled clotting activation, thrombosis, edema, local hypoxia, and ischemia are initiated after glycocalyx degradation in sepsis [67]. Thus, the use of eGC components such as biomarkers in sepsis may open new avenues for neonatal sepsis research. The PBR is one of these eGC components, which has demonstrated an area under the curve (AUC) of 0.778 in the case of hospital mortality in sepsis, especially after 24 h in ICU admission. It can be used as an indicator of loss of barrier function by positive correlation with ang-2 (rho ¼ 0.532, P ¼ 0.03), although it was not associated with other molecules such as lactate, SDC-1, angiopoietin-1, heparin-binding protein, or clinical scales such as the SOFA score and APACHE IV score [68].

Heightened levels of plasma SDC-1 and HA in septic patients indicate the disease severity and predict the development of disseminated intravascular coagulation (DIC) [69]. The levels of HA and SDC-1 increase in severe cases of sepsis and septic shock patients according to their levels, so HA and SDC-1 can be used to predict the progression of patients to septic shock. In addition, both biomarkers were found to decrease in survivors than in non-survivors, where receiver operating characteristic (ROC) curve analysis for mortality prediction had cut-off values of 441 ng/mL and 898 ng/mL for HA and SDC-1, respectively. Moreover, the specificity and negative predictive values were (90%/90%) and (86%/91%) for either HA and SDC-1, respectively [70]. The serum analysis of 100 patients in the intensive care unit (ICU), showed increased SDC-1 levels in non-survivors compared to survivors on day 1 and day 3 (*p* < 0.01). The levels of SDC-1 were associated with multiple organ dysfunction, dysregulation of the coagulation process, and renal failure, been correlated with the DIC score (rho = 0.33, *p* < 0.01). Moreover, if levels of SDC-1 were higher than 21.4 ng/mL, the patient recovery from a thrombocytopenia situation was delayed [71]. Another study confirmed that SDC-1 levels were associated with a high risk of organ dysfunction and mortality. Furthermore, the loss of eGC was also associated with acute respiratory distress syndrome (ARDS) in non-pulmonary organ dysfunction in patients with sepsis [72]. 

## 7. Neonatal Sepsis and Glycocalyx Sheddase Enzymes

**Figure 2:**
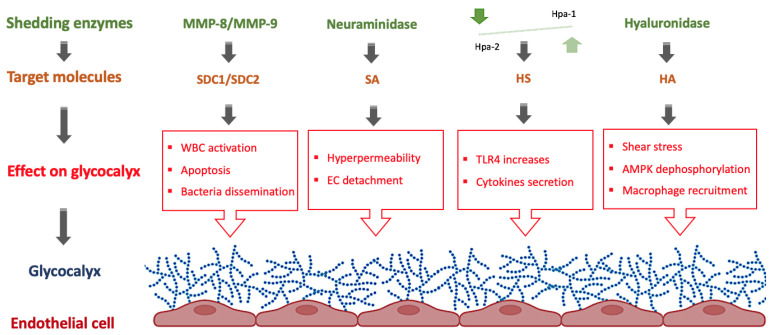
Role of shedding enzymes on the eGC in neonatal sepsis. AMPK: AMP-activated protein kinase, EC: Endothelial cells, HA: hyaluronan, Hpa: heparanase, HS: heparan sulfate, MMP: matrix metalloproteinases, SA: Sialic acid, SDC: syndecan, TLR: toll-like receptors, WBC: White blood cells. Figure created with BioRender.com.

During a septic episode in adults, the main enzymes involved in glycocalyx shedding (i.e., matrix metalloproteinases, hyaluronidase, and heparinase) are dysregulated. It would be conceivable that these enzymes may play a similar role in neonatal sepsis, and their function may be compromised during a septic episode in newborns. Despite this, limited research has been conducted to characterize the role of the glycocalyx and related enzymes in neonatal sepsis (Figure 2). 

Among the scarce existing research on this topic, there are few studies describing the implication of some key enzymes in neonatal sepsis, specifically in endothelial dysfunction. In one study developed by Achten et al., the serum levels of MMP-9 were determined in 23 newborns diagnosed with late-onset sepsis (LOS) by confirmed blood cultures, and MMP-9 levels were shown to be increased in septic neonates and downregulated in non-survivor patients [73]. Dreschers et al., by performing a serial of experiments using E. coli-infected neonatal monocytes, demonstrated the increase of MMP-9 in infected monocytes. Interestingly, MMP-9 activity was required to inhibit cell contact-dependent phagocytosis-induced cell death and therefore, it may be involved in long-term inflammation in newborns [74]. In a prospective study including 68 newborns with early-onset sepsis (EOS) and 83 newborns as a control group, it was demonstrated that IL-6, TNF-α, HSP70, PCT, and CRP together with MMP-8 can be used as a predictive biomarker of EOS. The sensitivity and specificity of MMP-8 reached 82.35% and 38.55%, respectively, and the AUC (95% CI) was 0.607 (0.524–0.685) [75]. A study performed on very low birth weight (VLBW) infants with blood culture-proven LOS showed the relationship between MMP-8 mRNA up-regulation and the increase in organ failure in pediatric patients, which was directly related to the decrease in patient survival [76]. In a study including 16 human placentas from newborns, Naeh et al. reported the level of heparanase was associated with differences in the gestational age and the birth method, where the high levels of heparanase were found in preterm vaginal birth and term cesarean deliveries as compared to term vaginal birth [77]. When heparanase expression was evaluated in placental tissue from nine preeclamptic patients and three healthy controls at term, it was found thst it is overexpressed in preeclamptic placentas compared to controls, confirming its feasible contribution to the development of preeclampsia [78]. These findings showed that heparanase has a role in preterm labor and preeclampsia, and high heparinase levels are a risk factor for newborn sepsis. The Hpa-1 activity also increased the EC Toll-like receptor 4 (TLR4)-mediated sensitivity to LPS. Heparanase 2 (Hpa-2), on the other hand, protects EC against Hpa-1-mediated damage by reducing TLR4 activation, cell signaling, and cytokine production [79]. In sepsis, Hpa-1 was increased while Hpa-2 was decreased. Moreover, according to Pape et al., Hpa-1 seems to be harmful while Hpa-2 may have a protective effect, suggesting that both enzymes may work as antagonists. In line with these results, it is noteworthy that in clinical practice, therapeutic plasma exchange allows the restoration of Hpa-1/Hpa-2 balance, so attenuating eGC breakdown [80].

Via hyaluronidase activity, the group B streptococcus (GBS) can reverse uterine immune responses without influencing inflammatory reactions, thus increasing the risk of ascending routes of infection in pregnancy, early labor, and fetal death [81]. The production of HA can be modulated by pro-inflammatory signaling pathways IL-1β, TNF-α, and TNF-β in EC via an NF-κB-dependent manner [82].

Increased vulnerability to neonatal sepsis has been linked to genomic polymorphisms that affect cytokine production and cell signaling pathways. According to Esposito et al., the *MMP16* gene was associated with increased susceptibility to developing neonatal sepsis [83]. However, although MMPs can participate in endothelial glycocalyx damage, no information about the role of MMP-16 in the eGC is yet available. 

In experiments performed in HUVEC challenged with LPS, the addition of MCTR1 downregulated heparanase expression, thus inhibiting the HS degradation [64]. In addition, results obtained by Zhang et al. in HUVEC revealed that hyaluronidase 2 and low shear stress activates the Na+-H+ exchanger induced AMPK dephosphorylation, contributing to glycocalyx injury, macrophage recruitment, and inflammation via HA degradation [84]. Delgadillo et al. used an enzymatic treatment on HUVEC cells to remove the eGC components HA and HS to generate a model for eGC damage. After enzymatic treatment, the authors observed that the individual removal of HA or HS did not affect eGC thickness and neutrophil adhesion. However, when HA and HS were removed simultaneously, eGC thickness was observed together with increased neutrophil adhesion to the endothelium and increased rates of fluid leakage [85].

## 8. Biomarkers for Neonatal Sepsis: Focus on Endothelial Glycocalyx

The endothelial glycocalyx is hyperactive in healthy preterm and term newborns, characterized by high expression of soluble sVCAM-1, sICAM-1, and sE-selectin. Therefore, alteration in the normal blood circulating levels of these proteins makes them good candidates for clinical use as diagnostic biomarkers in neonatal sepsis [86]. The hyperactive state of the EC is the consequence of the constitutive TNF-α production and has been linked to the hyperdynamic nature of systemic inflammatory response syndrome (SIRS) in neonates with the physiological trafficking of leukocytes to several organs and tissues [87]. In this regard, some authors demonstrated how *Pneumocystis carinii* infection can induce downregulation of TNF-α in the EC of neonatal mice models [88]. Focusing on the molecular mechanisms, it is established that the expression of adhesion molecules on the EC surface impairs the capacity of T-cell migration, and alters the host defense response to infection [89]. This molecular cascade lead the neonatal sepsis mice models to poor outcomes, and demonstrated the role of the vascular system and its components in the progression of neonatal sepsis [87]. A summary of the central components and enzymes related to the eGC during sepsis can be found in Table 1.

Other major modulators of the EC in neonates are the C and S proteins. As it is well known, breast milk is one of the most important anti-infective agents, of which up to 55% of the glycosaminoglycans (GAG) consist of C and S proteins. Protein C and protein S are glycoproteins involved in the natural physiological anticoagulant system, playing an important role in maintaining normal endothelial homeostasis [90]. In this regard, some authors found that these proteins can decrease bacterial invasion and translocation in cell culture. Specifically, C and S protein levels of 750 μg/mL can decrease up to 75% of *E. coli* invasion, avoiding translocation without affecting cell viability [91]. 

Similarly, other potential biomarkers for neonatal sepsis are the circulating levels of Inter-alpha inhibitor proteins (IαIp). IαIp is a protein family of endogenous serine protease inhibitors, found in human plasma, that modulate endogenous protease activity. Interestingly, therapies based on IαIp improved survival in both adult and neonatal mice sepsis models [92]. That means that plasmatic levels of IαIp could help clinicians and scientists not only to improve the current diagnosis and prognosis of neonatal sepsis but also open a new avenue of therapeutic approaches to prevent the deleterious effects that neonatal sepsis induce on the endothelium. Regarding the molecular mechanisms, IαIp proteins contain heavy and light polypeptide subunits, that make a covalent bond with the GAG chain, contributing to physiological and pathological activities, including stabilization of the extracellular matrix and inflammation [93]. Commonly, IαI is abundant in the circulation and can regulate the expression of cytokines and endothelial factors. In sepsis, IαIp correlates inversely with soluble sVCAM-1 and sICAM-1, which could indicate a direct role of IαIp in modulating the expression levels of VCAM-1 and ICAM-1 [94]. Regarding newborns, levels of IαIp in blood did not change with gestational age (24–42 weeks) and were similar to levels found in adults. Nonetheless, in neonatal sepsis caused by Klebsiella and Candida, the expression levels of IαIp decreased to a lesser extend compared with sepsis caused by *Staphylococcus epidermidis* [95]. In this regard, in a study including 573 newborns with sepsis, IαIp excelled as a biomarker for neonatal sepsis. Specifically, IαIp levels decreased in septic patients with an AUC of 0.94 (95% CI, 0.92–0.96, *p* < 0.0001), and the optimal cutoff value with the ROC curve was ≤177 mg/L (sensitivity, 89.5%; specificity, 99%; positive predictive value, 95%; negative predictive value, 98%) [96]. Unfortunately, as did occur with other biomarkers, IαIp was unable to differentiate systemic neonatal sepsis from necrotizing enterocolitis (NEC), a highly proinflammatory disease, but was applicable to differences between NEC/sepsis and non-sepsis [97,98]. In a mouse animal model, injection of IαIp modulated the immune response, and suppressed secretion of the proinflammatory cytokines such as TNF-α, improving the survival in mice models (~90%, *p* = 0.0159) in both live bacterial infections and with LPS-induced sepsis. This study concluded that IαIp is an important therapeutic adjuvant in neonatal sepsis [92]. 

Finally, another important molecule related to the endothelial glycocalyx is endocan. Endocan, a chondroitin/dermatan sulfate proteoglycan, is an important endothelial mediator, which has been found to be increased in SIRS and septic shock. In this regard, Zonda et al. reported the upregulation of endocan just after ICU admission of neonates with sepsis compared to the non-septic population. This proteoglycan remained high until the third day and returned to normal values on day 7. Specifically, endocan showed an AUC of 0.73 (*p* = 0.004, 95% CI = 0.597–0.871) [99]. Another study performed by Saldir et al. showed significantly increased endocan levels in the serum of neonates with LOS, where the AUC showed values of 0.80 (95% CI = 0.674–0.923, *p* < 0.001). Thus, endocan presents a good indicator for the diagnosis of LOS and monitoring therapy response thereafter [100]. Another study confirmed the correlation between higher leukocyte levels, CRP, IL-6, and endocan in the serum of neonates with LOS. Whereas, the level of endocan in non-surviving patients was higher at every time. Therefore, endocan is another interesting biomarker in LOS preterm infants [101].

**Table 1:** Potential biomarkers of the endothelial glycocalyx in neonatal sepsis.

Biomarkers	Biological Role	Changes in Neonatal Sepsis	AUC	Ref
Matrix metalloproteinase-9 (MMP-9)	Zinc-dependent proteinase, is released by various inflammatory cells, predominantly neutrophils and macrophages. Implicated in oxidative stress, inflammation and lung injury.	Augmented in septic neonates (LOS) and downregulated in non-survivor patients. In vitro, increased in *E. coli*-infected neonatal monocytes, and inhibit cell-contact-dependent phagocytosis-induced cell death and may help to reduce long-term inflammation in newborns through this mechanism.	Not described in the literature	[73,74,102,103]
Matrix metalloproteinase-8 MMP-8	A neutrophil-derived collagenase Degrade collagen type I. present in macrophages, fibroblasts, epithelial cells, and other immune cells. MMP-8 has non-collagen proteolytic targets, including pro-inflammatory chemokines. In sepsis, its levels were increased. MMP-8 promotes leukocyte adhesion to HUVECs.	In EOS increased with other molecules such as IL-6, TNF-α, HSP 70, PCT, and CRP. In VLBW with LOS the elevation of MMP-8 mRNA expression and activity in septic shock correlated with decreased survival and increased organ failure in pediatric patients and associated with TNF-α. MMP-8 is associated with poor prognosis in sepsis.	0.607 (95% CI = 0.524–0.685)	[75,76,104,105]
Inter-alpha inhibitor proteins IαIp	Serine protease, it is prominent among the histone-precipitated proteins. IαIP is composed to light and heavy chains (HC). In inflammation, IαIP interacts with TNF-stimulated gene six protein (TSG-6), which supports trans-esterification of HC to HA. In sepsis, IαIp inhibit granzymes and other proteases reducing their toxic proteolytic activity.	In NS the level of IαIp was decreased. Its levels were inversely correlated to 28-day mortality rates and illness severity.	0.94 (95% CI = 0.92–0.96, *p* < 0.0001)	[96,106,107]
Endocan	Endothelial mediator, it mainly inhibits leukocyte diapedesis rather than leukocyte rolling or adhesion to the endothelial cells both in vitro and in vivo.	Increased in NS and remained high until the third day, returning to their normal values on day 7. It is increased in the case of LOS. It is proven in the diagnosis of sepsis, and sepsis severity but its prognostic value was better compared with procalcitonin.	0.73 (95% CI = 0.597–0.871, *p* = 0.004) 0.80 (95% CI = 0.674–0.923, *p* < 0.001)	[99,100,108]

AUC: Area under the curve; CI: confidence interval.

## 9. Endothelial Glycocalyx and Neonatal Sepsis Therapy

**Figure 3:**
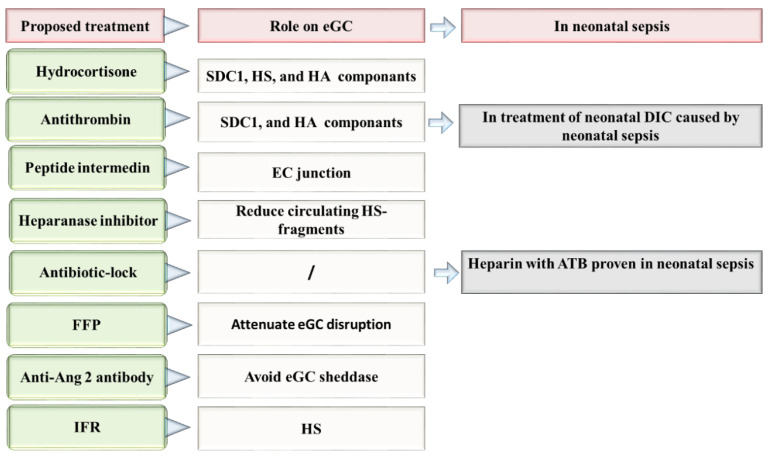
Proposed therapies directed to the repair of eGC in neonatal sepsis. Ang 2: Angiopoietin-2, ATB: antibiotic, DIC: disseminated intravascular coagulation, EC: Endothelial cells, eGC: Endothelial glycocalyx, FFP: fresh frozen plasma, HA: hyaluronan, HS: heparan sulfate, IFR: Intravenous fluid resuscitation, SDC-1: syndecan 1.

The eGC is altered and shedded in sepsis, affecting the normal endothelial homeostasis. Therefore, eGC components are potential biomarkers for early diagnosis and prognosis of sepsis, and their restoration may set the basis to design potential therapeutic strategies against sepsis (Figure 3) [109]. In this section, we describe some molecules and therapeutic strategies which can open new avenues in the designing of specific therapies against sepsis based on the recovery of the eGC.

It has been demonstrated that hypervolemia and hyperglycemia can be toxic to the glycocalyx. Some studies have explored the therapeutic effects of many molecules to avoid eGC damage. However, to date, all tested glycocalyx-based treatments have failed [110,111]. Among the molecules studied, hydrocortisone, besides its cytokine-suppressing effects, can increase effective circulating blood volume and systemic vascular resistance [112]. In fact, it has been found that hydrocortisone and antithrombin can preserve the eGC during inflammatory-mediated degradation initiated by TNF-α [113]. In experimental models, intravenous hydrocortisone reduced the shedding of glycocalyx components SDC-1, HS, and HA, and decreased the formation of extravascular edema [65]. In a rat sepsis model, the antithrombin-treatment downregulated the circulating levels of SDC-1 and HA, and improved leukocyte adhesion, and blood circulation [114]. In the neonatal period, hydrocortisone represents the third-line response to treat neonatal shock. However, its role as a potential treatment for neonatal septic shock has not been yet evaluated. Hydrocortisone treatment in neonatal period has been found able to increase systemic arterial pressure, reduce the heart rate, and the necessity to use vasoactive drugs in newborns [76,115]. In contrast, the use of antithrombin during neonatal sepsis remains uncertain [116]. Nonetheless, in a study performed by Hayato et al., the authors observed the efficacy of antithrombin to treat neonatal DIC occurring during neonatal sepsis [117]. 

The peptide intermedin, a calcitonin family member, plays the role of self-protective factor in sepsis. In a septic mice model, intermedin participates in the mechanisms of repairing the endothelial junction disruption. In addition, it decreases the responsiveness of inflammatory and macrophage infiltration, thus preventing organ injury and therefore increasing the survival of infected mice [118]. Nevertheless, as far as we are aware, no study has been published so far on intermedin as a therapeutic tool or molecule in neonatal sepsis.

The study of Schmidt et al. is based into the role of endothelial heparanase in the shedding of eGC in mice after LPS-induced sepsis. In this work, the authors noted that the mice pre-treated with heparin or the non-anticoagulant heparanase inhibitor N-desulfated/re-N-acetylated heparin avoid the LPS-induced eGC shedding, thus attenuating sepsis-induced inflammatory lung injury [119]. The use of low molecular weight heparin avoided thrombosis in neonates [120]. In newborns, it was observed a decrease of culture-positive catheter-related sepsis via heparin. Specifically, Birch et al., reported that adding 0.5 IU/mL of heparin to total parenteral nutrition was a very effective manner of reducing sepsis without any adverse complications [121]. Until now, low doses of heparin can reduce the risk of catheter obstruction, and maintenance of percutaneous central venous catheters, thus allowing successful sepsis therapy completion [122]. In addition, the association between heparin and vancomycin (vancomycin-lock) has prevented catheter-related sepsis in VLBW preterm neonates and reduced antibiotic exposure, without causing common complications, including hypoglycemia [123]. In general, all antibiotics combined with heparin “antibiotic-lock solution” appear to decreases the risk of catheter-related bloodstream infection in the neonatal population with a high efficacy [124]. However, despite the fact that heparin seems to improve further complications in septic cases, the existing studies do not elucidate heparin’s true mechanism of action on endothelial vascular cells, or particularly on eGC.

Intravenous fluid resuscitation, generally with crystalloids or some mineral salts, or other soluble molecules, is commonly used nowadays in sepsis treatment [125,126]. However, as a therapeutic strategy may induce iatrogenic endothelial injury. This idea is based on the results found by Hippensteel et al. who mentioned the relation between the volume of intravenous fluids injected and plasma HS during resuscitation. Regardless of the sepsis severity and patient age, every liter of intravenous fluids can increase up to 200 ng/mL of circulating HS. Thus overaggressive fluid therapy can induce glycocalyx degradation [127]. Therefore, there exist undesirable effects produced by the administration of fluid resuscitation in VLBW infants. In fact, there is evidence that after two days of birth, the use of fluid resuscitation can increase the risk of chronic lung disease, patent ductus arteriosus, intraventricular hemorrhage, and the increase of risk of death [128]. To our knowledge, there are no research published so far about the role of fluid resuscitation in the treatment regimen of neonatal sepsis. Nonetheless, the results published by Bakshi et al., urge caution regarding the use of fluid resuscitation in newborns with sepsis until new studies provide more data on this issue. 

Alternatively, it has been proposed that the use of fresh frozen plasma (FFP) containing albumin to attenuate eGC breakdown [127]. Unfortunately, the benefits or possible side effects of FFP on glycocalyx integrity in sepsis have not been yet studied. Therefore, further efforts and clinical research is needed to demonstrate how these feasible therapies may improve treatment options in neonatal sepsis [49]. Acunas et al., observed that the administration of FFP and gamma-globulin can modulate humoral immunity in neonatal sepsis and induce the increases of immunoglobulins IgA, IgM, and C4 concentrations. Importantly, the authors observed that the likelihood of survival augmented in septic patients after the administration of FFP and gamma-globulin [129]. However, these results should be taken with caution because the use of only FFP did not improve the overall state of neonates diagnosed with neonatal sepsis [130]. In any case, further research evaluating how FFP treatment can mitigate endothelial injury in sepsis, particularly by avoiding the eGC layer, would improve the outcome in neonatal sepsis. This may help to further clarify the potential therapeutic possibilities of this kind of treatment and avoid the transfusion of adverse reactions [131].

In sepsis, HS-fragments released into the bloodstream act as strongly damage-associated molecular patterns, inducing pro-inflammatory phenotypes through TLR4-dependent pathways. Reducing circulating HS fragments represents a new therapeutic strategy against sepsis [59]. Similarly, the administration of heparanase inhibitors for 2 h during early sepsis in mice models attenuated the loss of glomerular filtration rate and attenuated the serum levels of IL-10 [132]. However, despite the prominent role of the endothelial glycocalyx in vascular homeostasis, its importance in some therapies, such as intravenous fluid resuscitation therapies, is still largely unknown. 

The pathway Angiopoietin-Tie2 was implicated in bacteremia and mortality in neonatal sepsis [133]. Ang-2 was demonstrated to reduce the expression of receptor Tie2 in the EC, to increase endothelial permeability, and therefore contribute to edema formation in vivo [52]. In mouse models of sepsis, the use of an anti-Ang2 antibody ABTAA (ANG2-binding and Tie2-activating antibody) aids in vascular protection, via reducing cytokine storm, avoiding eGC sheddase, and vascular leakage [134]. These results make the role of the Ang-Tie2 axis feasible in sepsis. Particularly, low Ang-1 and high levels of Ang-2, as well as a high Ang-2/Ang-1 protein ratio in serum have been previously associated with EOS in Surinamese newborns [135]. Therefore, because angiopoietins may play a role in the vascular pathophysiology of EOS, it is feasible that the Tie2 activation may ameliorate sepsis progression. If this hypothesis is demonstrated, the control of the ratio Ang-Tie2 can become a sepsis-specific treatment via restoring the eGC and the microvascular barrier, thus accelerating mechanisms mediating angiogenic repair.

## 10. Conclusions

The eGC is one of the most important vascular integrity regulators, playing a central role in vascular homeostasis. However, despite their prominent role, most research has been focused on adults and only a few studies have been done on neonates during the last decades. Therefore, the function of the eGC in newborns, and particularly its importance during neonatal sepsis, is still widely unknown. 

Sepsis is characterized by a pro-inflammatory response during the first stages of disease, inducing the well-known “cytokine storm”, that damage different organs and tissues, being the endothelium the first one, since it is covering the blood vessels and the different tissues and organs. That means the endothelium is one of the most damaged organs during sepsis. So, its protection and recovery are essential for a normal healthy status, especially in newborns that suffered a sepsis episode. 

Biomarkers that help clinicians to diagnose neonatal sepsis as early as possible would help to avoid the disease progression, as well as to reduce endothelial damage, which can produce eventual pathogenic processes related to endothelium. However, unfortunately, neonatal sepsis is still hard to diagnose, and there is limited possibility for clinicians to differentiate between sepsis and other diseases that share similar clinical phenotypes. Moreover, as if that were not enough, the treatments are also very limited, and yet sepsis neonatal is a critical condition in newborns, not only because of the mortality but mainly due to the associated comorbidities.

As for the most important biomarkers, circulating levels of MMP-9 stand out in LOS. MMP-9 has been found to be upregulated in septic neonates and downregulated in non-survivor patients. Similarly, MMP-8 correlates with the increment of the risk for organ failure in children. Moreover, IαIp is another exciting candidate among the different biomarkers, since it can work as both, a biomarker and therapeutic adjuvant in neonatal sepsis.

Developing novel therapeutic strategies based on novel anti-inflammatory molecules that can interact with heparanase and control the level of HS-fragments may be another interesting approach. The Tie2 targeting system appears to have a relevant role, despite its specific function in the vascular barrier needing to be fully characterized. In this regard, the use of fluid resuscitation as a therapeutic tool also needs more studies to evaluate their effect on the eGC integrity and normal function. 

In this work, we tried to shed light on the important role of the eGC in the early diagnosis and prognosis of newborn sepsis, and the potential of developing new therapeutic strategies taking as a central player in the neonatal eGC. 

## Data Availability

Not applicable.
